# ‘*It’s so heavy on my mind’*: The lived experience of diabetes in pregnancy and postpartum mother and infant lifestyle

**DOI:** 10.1186/s12884-024-06723-5

**Published:** 2024-09-10

**Authors:** Jacob Morton-Jones, Jennifer Brenton-Peters, Lisa Blake, Siniva Sinclair, Julienne Faletau, Eva Takinui, Elizabeth Lewis-Hills, Charlotte Oyston

**Affiliations:** 1 Health New Zealand - Te Whatu Ora Counties Manukau District, Auckland, New Zealand; 2 Waikato-Tainui, New Zealand; 3 Regional Diabetes Service, Health New Zealand - Te Whatu Ora Waikato District, Hamilton, New Zealand; 4 Ngaati Whaatua, New Zealand; 5 Department of Obstetrics and Gynaecology, Health New Zealand - Te Whatu Ora Counties Manukau District, Auckland, New Zealand; 6 Department of Obstetrics and Gynaecology, Faculty of Medical and Health Sciences, University of Auckland, Auckland, New Zealand

**Keywords:** Diabetes, Pregnancy, Antenatal, Postpartum

## Abstract

**Background:**

Diabetes in pregnancy is associated with short and long-term adverse health outcomes for mothers and babies. The condition disproportionately impacts Pacific, Maaori, and Indian pregnancies. Those with diabetes are offered intensive support during pregnancy, but in many centres, no additional support following birth beyond routine care. The aim of this study was to determine the postpartum needs for mothers and *whaanau* (family) impacted by diabetes in pregnancy, to inform co-design of a new service to improve metabolic and developmental outcomes for infants.

**Methods:**

Pregnancies complicated by diabetes were identified using a local diabetes registry. Mothers with a diagnosis of GDM or T2DM and gave birth between January 2022 -June 2022 were eligible for the study. A total of 19 parents (18 mothers, 1 father) were interviewed. Participants primarily identified as Maaori (6), Pacific (6), Indian (3), Asian (2), and New Zealand European (2). Interviews took place between December 2022 and February 2023, between 5-13 months post-pregnancy. Interviews facilitated by an externally contracted local Pacific mother, with a professional background in social work were conducted using *Koorero* and *Talanoa*, Maaori and Pacific methods of qualitative data collection, to capture the lived experiences of participants. A multidisciplinary group reviewed and coded interview transcripts. Themes were presented back to participants and stakeholders for feedback and refinement. Four over-arching themes were illustrated with exemplar quotes.

**Results:**

Four themes described the importance of 1) Finding a balance between what is “best practice” and what is best for me, my baby and my *whaanau*, 2) The need for individualised and culturally appropriate care, 3) Centrality of *whaanau* and family to the pregnancy and postpartum journey, 4) The pervasive negative impact of diabetes on *taha hinegaro* (well-being) beyond childbirth.

**Conclusion:**

People who are affected by diabetes in pregnancy have ongoing physical, psychological, and social needs. A holistic, whaanau-centred approach is required to ensure optimal health and well-being outcomes of mothers, infants and *whaanau*. The findings of this study will inform a new targeted model of care for infants and *whaanau* affected by diabetes in pregnancy.

## Introduction

The first 1,000 days of a child’s life creates the foundation for health and well-being [1]. Pregnancy complications such as diabetes, preeclampsia, large-for gestational age and preterm birth are associated with longer term health conditions for both mothers and babies [2, 3]. Supporting health and wellness for people with pregnancy complications, their children, and families over the first 1,000 days could ameliorate the impact these conditions have [4–7].

Gestational Diabetes Mellitus (GDM) is the most common pregnancy complication [8]. With the growing prevalence of pre-diabetes and Type 2 Diabetes Mellitus (T2DM) in reproductive women [9], the risk of Diabetes in Pregnancy (DiP) increases. This is observed within our region (Counties Manukau, Auckland, Aotearoa New Zealand), where rates of DiP have more than quadrupled from 3% of births in 2006 to 14% in 2022 [10]. The burden of diabetes falls inequitably on Pacific, Indian, and Maaori women (38%, 19% and 17% of our local DiP consumers) [11], with these ethnicities also experiencing worse health outcomes related to diabetes, both within and outside of pregnancy [11–13].

Up to 50% of women with pregnancies complicated by GDM, go on to develop T2DM [14]. Children born to those with DiP have increased adiposity, and poorer developmental outcomes during the first 1,000 days [15, 16]. Although the mechanism explaining these associations is not well understood, there are differences in postnatal environments that may contribute to these differences, and importantly, might be amenable to change [15]. Examples of these include shorter duration of breast-feeding, activity, sleep practices, early complementary food introduction, diabetes management and maternal health and well-being [15–17].

During pregnancy women with diabetes have intensive follow-up and support. Post birth, despite the availability of several community services supporting health behaviours, these services are fragmented from routine care, relying on individuals pursuing support and / or health care provider recognition of need and then referral. This is an opportune time to support the development and maintenance of healthy and preventative behaviours amongst mothers with diabetes in pregnancy, which would have a flow on effect to their babies and potential future pregnancies [18]. To our knowledge, in Aotearoa New Zealand, there are no tailored secondary care postnatal services that provide wraparound support and connection with primary care to people who have / have had DiP. This gap in care is of concern, particularly given this period of growth lays the foundations for optimal metabolic health outcomes in children [2].

Approaching healthcare from the world view of the priority population is important in reducing health inequities [19]. Understanding *“Hauora”*, the Maaori philosophy of holistic health and well-being [20, 21], is key when approaching healthcare from a Maaori view. *Te Whare Tapa Wha* is a model developed to understand Maaori approach to health and wellbeing [21]. *Te Whare Tapa Wha* is represented by a whare (house) with four dimensions of health depicted as the walls, that must be in balance to achieve health and well-being. These four dimensions include [21]: *taha wairua* (spiritual health), *taha hinengaro* (mental health), *taha tinana* (physical health) and *taha whaanau* (family health) [21]. Pacific literature describes a similar holistic approach to health and wellness, and uses models, such as *Fonofale* [22], to illustrate these concepts.

We hypothesise that a culturally appropriate, wraparound service to support those who have a pregnancy complicated by diabetes, and their families / *whaanau*, might improve longer term maternal and child health outcomes. This service would align with the holistic Maaori philosophy of health and well-being “*Hauora*” to ensure equitable service access and outcomes are achieved. Such a service would weave together community, primary and secondary care services, to support families across the first 1,000 days of their child’s development. To prevent the perpetuation or broadening of health inequity, any new service must be developed in partnership with those who have experienced DiP and acknowledge the contributing factors of social determinants of health, environment, economics, genetics, stigma, and bias [23, 24].

The aim of this study is to explore and summarise the pregnancy and postpartum lived experiences of mothers affected by diabetes in pregnancy. The findings will inform and tailor a model of care which aims to improve metabolic and developmental outcomes for children born to mothers affected by DiP.

## Methods

### Ethics

This study was approved by the Auckland Health Research Ethics Committee (AH24822).

### Methodology

A qualitative exploratory research methodology [25] was used to capture the lived experiences of mothers / parents affected by DiP. A phenomenological approach [26] was used to investigate the experiences of our participants and explore their thoughts and understandings of key health indicators within the first 1,000 days.

### Recruitment

Mothers who had given birth between January 2022 to June 2022 were recruited using the Counties Manukau DiP registry. Our prioritised population was defined by mothers who identified with ethnicities inequitably represented in diabetes in pregnancy prevalence and poor health outcomes statistics (Maaori, Indian and Pacific ethnicities). A purposive approach by reported ethnicity was used to ensure equitable representation of prioritised populations. Potential participants were contacted by telephone, provided with information on the study and asked if they would be interested in participating in an interview. Mothers were informed at this time their partners and other *whaanau* members were also invited to be present and participate in the interview. If the mother agreed to participate, signed informed consent was obtained through a participant information sheet and consent form; this was also completed by any partners / other *whaanau* who agreed to participate in the study. It is noted contacted participants identified as women / mothers.

### Inclusion / Exclusion criteria

Mothers who had experienced stillbirth or neonatal death, those with Type 1 Diabetes Mellitus and mothers living outside the Counties Manukau catchment at the time of birth were not approached. Due to the nature of the study design and limitation in interpreting services, those who were not able to participate in an interview conducted in English were excluded. No interpreters were available for this study. While we understand it has substantial limitations, we gathered data from English speaking patients as a starting point with awareness that further research and improvement can involve non-English speaking participants. Furthermore, as English was the only common language of the research team and it was determined interpretation of translated data from potentially multiple languages could not be done so with validity.

### Data collection

A semi structured interview guide was developed by a multidisciplinary research team consisting of a Paediatric Dietician (Male / New Zealand European), Co-design Advisor (Female / New Zealand European), Public Health Physician (Female / Samoan), Obstetrician (Female / New Zealand European), Pacific Health Researcher and Academic (Female / Tongan) and Maaori Health Representative (Female / Maaori). The research team represents professional expertise in diabetes in pregnancy management, prediabetes research, Pacific diabetes research, infant nutrition, qualitative and quantitative diabetes / diabetes in pregnancy research, co-design methodology, and personal lived experience with family / *whaanau* diagnosed with diabetes in pregnancy. The interview guide was developed based on five areas of infant and maternal lifestyle that are key drivers of early metabolic health and wellbeing. These included breast / bottle feeding, complementary (solids) feeding, infant sleep habits, infant physical activity and screen time, and maternal wellbeing questions.

Interviews took place between December 2022 and February 2023, between 5-13 months post-pregnancy. *Koorero* (*Kaupapa* Maaori [27]) and *Talanoa* (Pacific groups [28]) methods of qualitative data collection were applied in this study. *Koorero* draws on oral tradition and cultural narratives to express experiences as Maaori [27]. *Talanoa* is underpinned by cultural values of respect, kindness, being culturally versed, showing compassion and reciprocity associated with Pacific ways of engagement and dialogue. The use of *Koorero* and *Talanoa* was conducted by a contracted interviewer, who was a local Pacific mother with a professional background in social work. A safe space was provided for mothers and their *whaanau* to share their lived experiences and provide valuable insights into the effectiveness of current healthcare services. The interviewer had no connection to the DIP service or had any prior relationship with the study participants.

### Data analysis

All interviews were audio recorded and transcribed verbatim, with potentially identifying details redacted. They were then presented to all members of the Study Working Group (JMJ, JBP, SS, LB, JF, CO, ET), who familiarised themselves with the data through line-by-line reading. An inductive approach was undertaken for the initial codes identified by the Group. These were discussed in *hui* (meeting) and matched to the five focus areas. This *hui* was facilitated by the Co-Design Advisor (LB) and contracted interviewer (invited to ensure participant voice / intention was accurately interpreted from the transcripts). Twenty-one codes were categorised by the five areas of focus. If any codes were disputed among the Study Working Group, coding was to be determined / clarified by the Co-Design Advisor (LB); it is noted that no disputes for coding occurred.

Every effort was made by the Study Working Group to ensure that coding was reflective of participant data. The multidisciplinary and multi-ethnic nature of the Study Working Group was to ensure that data interpretation and coding remained as unbiased as possible and that participants' views and intentions were accurately captured.

The final overarching themes were determined by the study leads (JMJ, JBP, CO), and approved by the study governance group (see acknowledgements).

### Participant feed-back

Themes were presented back *kanohi ki te kanohi* (face to face) to the original participants and their *whaanau* in a group meeting at a local community facility. Participants and their *whaanau* were invited to reflect on the coding and thematic analysis and provide feedback to ensure themes accurately represented their lived experiences. All original participants were invited, of which 11 attended with their children and wider *whaanau* members (*n* = 38). Poster sheets displaying summaries of codes and initial themes for each of the five focus areas were displayed. Participants were encouraged to comment on or add to existing codes and themes. This follow-up *hui* was also attended by members of the Study Working Group and the contracted interviewer.

Four overarching themes were described (as above) to summarise themes across all sections. These final themes are described in the results section with exemplar quotes drawn from across the interviews to illustrate each theme.

## Results

### Participants

**Fig. 1:**
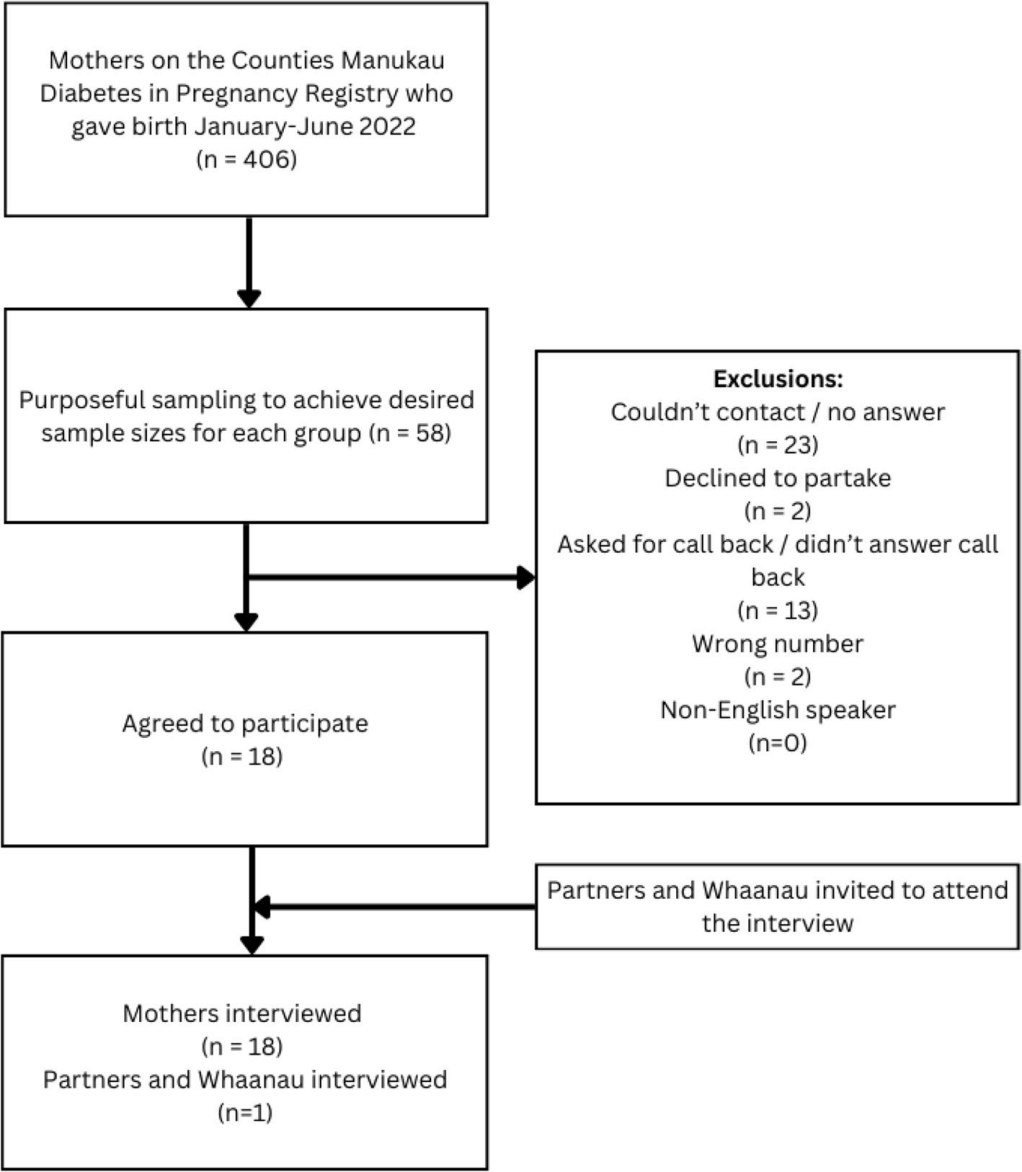
Consort diagram – Participant recruitment

Of the 40 women we successfully contacted, eighteen consented to participate (*n* = 18). The woman’s partners and *whaanau* were also invited to participate in the study at this time. One partner / father agree to attend the interview (*n* = 1) (see Fig. 1). The ethnicity of those interviewed were Maaori (32%; *n* = 6), Pacific (32%; *n* = 6), Indian (16%; *n* = 3), Asian (10%; *n* = 2 (1 mother, 1 partner/father), and Zealand European (10%; *n *= 2).

### Themes

A total of 21 sub-themes were identified from the five areas of focus (early feeding environments, complementary feeding and first foods, infant activity levels and screen time, sleep practices and diabetes in pregnancy experience).

**Fig. 2:**
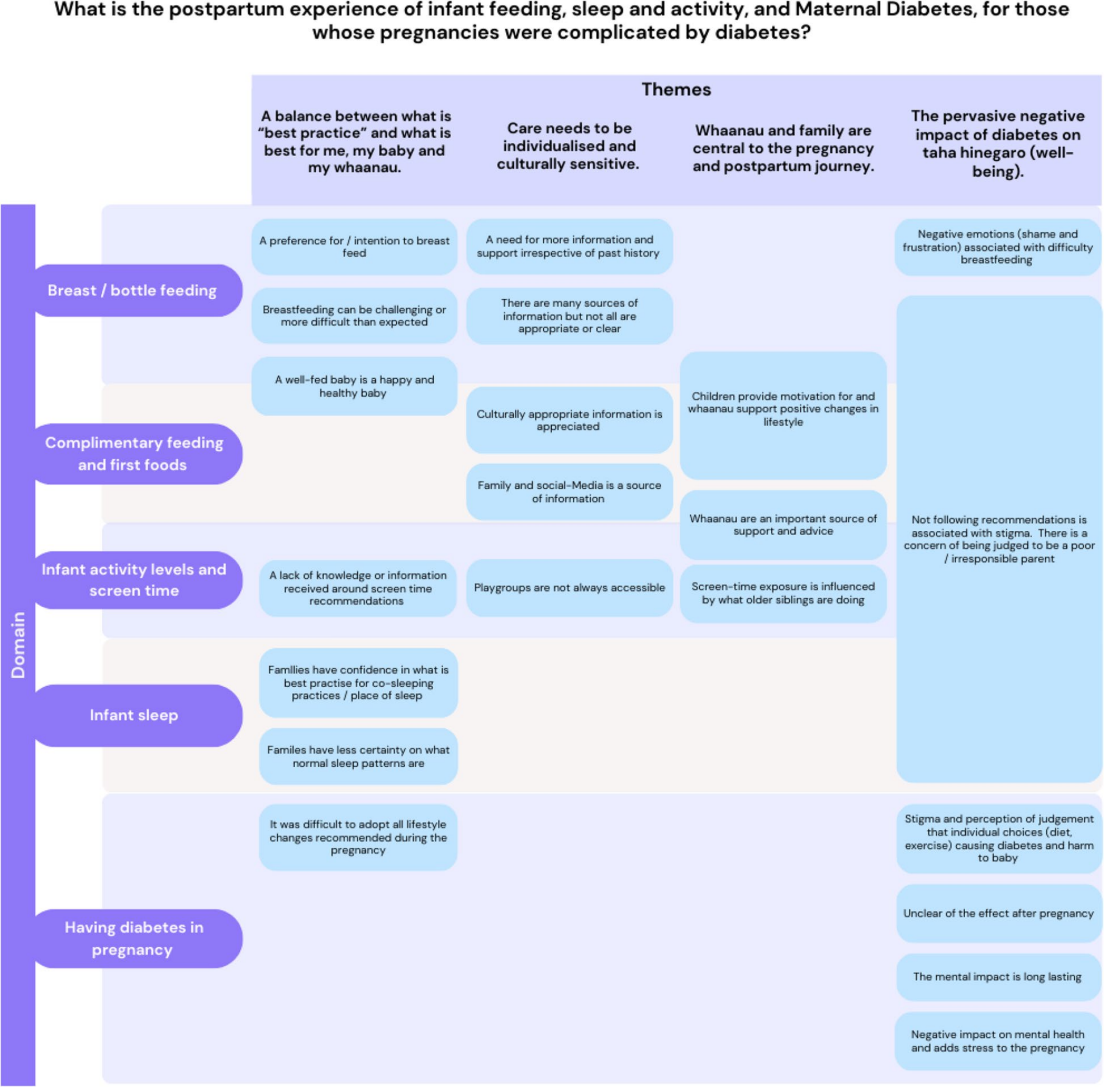
Coding diagram – Thematic Analysis

These sub-themes were organised into four overarching themes, which are illustrated in Fig. 2, and described below.

1. Finding a balance between what is “best practice” and what is best for me, my baby and my *whaanau*.

Participants were able to describe best practice advice that had been provided by health professionals but voiced a range of situations where this advice was difficult / impractical to follow, or did not address their needs at the time. In some cases, this was not due to a lack of information or a lack of understanding, but due to individual preference or situation conflicting with what was advised. For example, when asked if they were still affected by diabetes post-birth, one participant responded*:*

"I haven’t, I haven’t been for a blood test or anything but I really need to like my Doctors have sent me to, trying to get me to the lab to get blood tests done” (Maaori, P01)

In many cases, individuals agreed with what was advised, but due to their circumstances felt unable to continue along a recommended path. The most obvious examples of this occurred when discussing breastfeeding and use of formula. All parents interviewed had breastfeeding as their preferred option for feeding their children, *“My first preference to give breastfeeding [a go],” (Indian, P15),* with many parents noting the benefits of breastfeeding such as bonding, natural nutrients, cost and ease of feeding.

“Like my milk is medicine for her so if she was to get sick it will be easier for her to get better faster” (Maaori, P01)

However, often difficulties with establishing / maintaining breastfeeding, or other complications resulted in the introduction of either a bottle or infant formula, rather than breastfeeding.

“So in the end I just said mate you know what, I’m just going to bottle feed” (Maaori, P02)

“My milk supply wasn’t coming in strong, so my daughter was losing weight and I was then encouraged from a GP that is, maybe I need *to do* a bit of formula.” (Pacific, P08)

“When I had her it was covid and I tested positive so I had to stay away for 2 weeks so I kind of like faded out of breastfeeding.” (Maaori, P01)

Other examples of where best practice were noted but not followed included starting complementary feeding and sleep practices.

“I kind of put my baby on solids before they said.” (Maaori / Pacific, P16)

“He sleeps in our room sometimes, and then we’ll co-sleep, or in the cot.” (New Zealand European, P05)

The results further demonstrate the decision to choose alternatives to what was recommended could be associated with a sense of shame, failure, or concern about being perceived to be not doing the best for their child or family.

“That’s why I didn’t tell anyone put my baby on pureed food.” (Maaori / Pacific, P16)

“It felt like I was letting my baby down because I didn’t, because I couldn’t provide enough milk.” (Maaori, P02)

2) Care needs to be individualised and culturally sensitive.

Participants valued care that was individualised to their physical and cultural needs. Mothers felt frustrated when health professionals made assumptions about needs, for example, one mother described with her second child a lack of support and assistance establishing breastfeeding during her stay on the birthing unit.

*“I basically had to teach myself [breastfeeding] again*” (*Maaori / Pacific, P16).*

Within the postpartum hospital admission, there was a sense of being rushed through, leaving some mothers feeling neglected or unimportant.

“Like you talk about the support I think at the hospital that’s what they did, they gave me a little short video. I just felt like it was kind of rushed.” (Maaori, P10)

The lack of perceived support in hospital was compounded by observations that staff were busy, and under pressure, with some mothers acknowledging the COVID19 Pandemic may have influenced their care. Regardless, mothers expressed not wanting to be a burden with their needs.

“It was busy and covid and all that but maybe once they came over and I felt like if I asked them again they’d be like well we already taught you, learn. So I just fed everything and cried in the room.” (Maaori / Pacific, P16).

Mothers reported COVID19 influenced the way care was delivered antenatally, whereby most communication was conducted remotely or via videoconferencing.

“The support that I got because it was covid, we had to do zoom meetings and it was a zoom meeting, talking about what gestational diabetes was… I enjoyed the zoom meetings, she went through everything, all the zoom questions were answered.” (Maaori, P10).

In post-partum care, however, only one participants cited COVID19 influencing post-partum care, due to personal uncertainty around contraction of the viral illness and its potential on-going impact *“because of the unknown” (Asian, GM13),* leading to a self- imposed avoidance of social events.

“We’ve had to avoid you know social events so we reduce the risk of covid” (Asian, P13)

Throughout all *koorero / talanoa* it was evident that individuals valued care or information that considered their cultural identity or heritage. This was particularly evident when considering food and feeding. In those interviewed, some felt they were given choices that were appropriate for their cultural needs. For example, some Pacific mothers reported complementary food advice that incorporated culturally appropriate foods:

“Yes they do, they do, she did give me options. She gave me the paper and she said when baby’s turning 9 months, you can give her your cultural foods…. She suggests that I wait until he’s 9 months or 10 months, he’s more ready to take the Tongan food” (Pacific, P18),

“Yes, definitely gave me a lot of options… open my eyes to different variety cause usually like we think of our culture like we used to think of like [laughs] karo, potato, and things like that…I wouldn’t have fruit together with like Taro you know [laughs] for baby we can.” (Pacific, P07).

In contrast, Maaori mothers did not receive advice on feeding cultural foods,

*“No, I didn’t hear anything about cultural foods.” (Maaori, P04),* even though this was desired “*… If we do struggle around what to feed our kids, we want to look at what we can in a cultural aspect.” (Maaori, P10).*

Some mothers received mainstream advice that encouraged food choices that were unfamiliar to their families usual eating habits, which made it difficult or inappropriate to follow.

*“We would have to buy extra foods than we normally would, wouldn’t be foods that we usually would have*” (Maaori / Pacific, P14).

While others utilised digital technology and social media to source information when it was not readily available from a healthcare professional, or where family members were not directly accessible.

“Tik Tok is one of the best sources I can say from my personal experiences like from pregnancy until baby’s born til I started food.” (Indian, P03)

“Yup social media as well, I had a look on Google to see some ideas for picky eaters.” (Maaori, P10)

Several mothers concluded that advice around culturally relevant food suggestions or practices relevant to them had been missing from their postpartum journey, and is a key area that could be improved.

“*I think the information that we found, especially if it’s culturally for us Maaori or Pacific Islanders then if we do struggle around what to feed our kids, we want to look at what we can in a cultural aspect.” (Maaori, P10)*.

3) *Whaanau* and family are central to the pregnancy and postpartum journey.

Participants described the importance of *whaanau* / family support networks. Support was apparent in several ways. Infant and early childhood care was often shared amongst *whaanau* / family members, including fathers, grandmothers, aunties, and siblings.

“You know I’m blessed to have my husband who you know when he does see that I’m feeling that way he steps up big time, or even my parents and my sisters as well so I’ve got a really good support system.” (Pacific, P08).

Participants expressed frustration that involvement of postpartum support services often was directed toward the mother, and engagement with their spouse or other important family members was limited.

“I would say not helpful, it would have been really nice to sort of engage with my husband and the times they would come to my home and engage with my family, cause a lot of them would come to my home and just to make them feel like you know they have an important part in this as well you know so that would have been good yeah that would have been good.” (Pacific, P08).

“I think it would have been good to have my husband there because it just kind of feels like you’re in blood sugar’s high on this day,’ or ‘you’ve had three high sugars in one week’.” (New Zealand European, P05).

New parents looked to *whaanau* and family as first sources of advice.

“So my mums been a big influence when I first… cause I was 16 when I had my first baby so she helped me. I don’t know she guided me in the beginning so my mum and my grandmother.” (Maaori, P01)

Family, particularly the presence of older children also influence health related behaviours. Siblings were important for stimulating activity and physical play, “*Oh interaction with her brother is right throughout the whole day”* (Pacific, P08) and older siblings also appeared to be a contributor to early exposure to screens, *“Screen time is when my daughter comes after daycare then she’ll watch TV, then that’s the time that he’ll watch.” (Indian, P03).* Considering the needs and preferences of the whole family was also important in starting first foods “*Yeah [more culturally appropriate information] that would have been helpful cause what my families telling me is oh we don’t eat that kind of stuff in this house” (Maaori / Pacific, P16).*

4) The pervasive negative impact of diabetes on *taha hinegaro* (well-being) beyond childbirth.

The diagnosis of diabetes during pregnancy was itself a significant stress which persisted for months after the baby’s birth, even when repeat testing had shown the mother’s diabetes had resolved.

“It’s so heavy on my mind [laugh] because I feel like it’s something I can manage, it’s just sometimes I’m just like I just sort of chuck it out of my mind.” (Pacific, P08)

Women described personal guilt about the impact on their child’s current and future health.

“*I felt I let myself and the baby down. What did I do wrong?” (Maaori, P10).*

There was also concern about the potential for diabetes to reoccur and the impact that might have on their health going forward.

“I think it’s just scary now thinking that baby might end up getting it or any of my children and not only that, like, them losing me suddenly kind of thing due to diabetes.” (Pacific, P07)

Mothers shared the stigma associated with DiP and how it hindered their ability to talk about the care they needed.

“It’d find that the people I talked to just assume it’s because I was eating real crap food beforehand, and it’s like this big stigma about the fact that, yea, like I would say ‘yea I had gestational diabetes’ and people were like ‘oh well I eat healthy, so that’s why I couldn’t have it’ and it’s more like, it’s real hard to kinda, and if you try to correct they don’t really want to listen. They just assume that it’s because of how you’re eating.” (New Zealand European, P05).

Husbands or spousal partners were identified as particularly important for supporting pregnant mothers throughout pregnancy and beyond.

“I’m happy cause my husband was very supportive, he’s kept telling me it wasn’t my fault and that we’re in this together and most of the time he’s telling me not to hide my feelings cause I, cause I have depression on my older one and I mostly hide my emotional.” (Pacific, P18).

“No no, no it’s just my husband was there only to put me you know in stress free moment” (Indian, P03)

While mothers stressed the impact diabetes in pregnancy had on their *taha hingaero*, those who had had a continuous relationship with their midwife or a postpartum health provider voice positive affirmation of their services during pregnancy and after childbirth,

“Yup, we had a diabetes midwife, and we could call her if we needed her. I think there was a number you could call any time you were concerned about anything that was after-hours too which was really good.” (New Zealand European, P05).

## Discussion

Children born to pregnancies complicated by diabetes have an increased risk of non-communicable disease. Establishing a healthy lifestyle from infancy may ameliorate some of this risk, but there is currently no coordinated means of supporting health behaviours in the community. We have thematically analysed the postpartum experience for those with a pregnancy impacted by diabetes, to inform the design of a new postpartum support service. Our findings illustrated in Fig. 2 demonstrate a need for individualised care that is culturally sensitive, *whaanau* inclusive, and sensitive to the negative impact that diabetes has on four pillars of health as described by *Te Whare Tapa Wha*, particularly that of *taha hinegaro* (well-being) [21].

Our first theme described how participants were aware that they “should” provide care for their baby in certain ways, for example the benefits of breastfeeding, safe sleep practices, and when to start first foods. However, there were often barriers to following through, for example, mastitis, suboptimal infant weight gain, or a perception that an alternative way would be better for their family. Choice or inability to follow recommended practice was often experienced as shameful or distressing [24, 29]. Best Practice guidelines and preventative strategies should be designed with end-users (patients and families) in mind, but unfortunately these do not always consider individual *whaanau* circumstances, or preferences for care [30, 31]. Indigenous world views are holistic and include whakapapa (genealogy) impacted by lived-experiences, history, and changing environments [30]. While it is reassuring that important public health messages (breast-feeding, safe sleep, complementary foods) are acknowledged and understood, our findings suggest a need for practice / advice to account for, and adapt to parent / *whaanau* needs, or establish more effective ways of supporting parents and *whaanau* to overcome barriers to care. It is important to continue the promotion of these messages, but need to be conscious of potential harm (shame, alienation) that may come from focusing on promotion of these messages without genuinely supporting families to achieve them. This is particularly important as women are likely to be experiencing feelings of shame and failure relating to their diagnosis of diabetes, as described in our fourth theme. Providing ongoing care and the safest alternatives for those who are unable to meet medical best practise is important, in addition to maintaining advice and education about best practice.

While the COVID19 pandemic did influence antenatal care (as described in theme two), most participants did not cite the pandemic influencing post-partum care. The acceptance of tele-health as a method of communication during the antenatal period was perceived positively by participants, which is consistent with international literature [32]. However, many participants describe a lack of individualised support in their postpartum journey, with support being fragmented, insufficient, or ill-aligned with their cultural needs. Recent studies within our region demonstrate the success and value of continuity of care models for diabetes pregnancy care [33]. One potential solution is to extend continuity care models from pregnancy through the puerperium into the two years of postnatal life. While continued midwifery input would not be feasible beyond 6 weeks (due to widespread workforce shortages), other appropriately trained non-clinical health workers could fill this role.

Using non-clinical staff and digital technology, such as apps and social media, to promote health behaviours has been successful locally in pregnancy care. For instance, a dietary intervention delivered by a community health worker (a non-clinical worker of Pacific or Māori ethnic background, experienced in engaging hard-to-reach women in maternity care) with a Certificate of Pacific Nutrition, compared to the routine dietary advice had a significant reduction in total gestational weight gain of women affected by GDM living within Counties Manukau [34]. The difference was attributed to the community health workers’ ability to meet women at their desired location and provide a culturally tailored intervention. Similarly, the success of a co-designed ‘Good Start’ programme was attributed to its cultural tailoring, with the participation of Multicultural Health Workers who had lived experience and inherent cultural knowledge, specifically those identifying as Maori or Pacific living in Australia [35]. Digital technology and social media can assist with individualisation and meet mothers’ information needs on demand. However, similar to what was found in other studies [36], our study confirmed that social media is better received as a supplement to care, not as a replacement for personalised care.

The approach and delivery of health care matters. Collaborative, holistic care is essential when providing to those with stigmatised conditions such as diabetes and body size [37, 38]. Our participants identified a deficit of information and support that considered their culture / heritage; this was most noticeable in starting first foods (theme 2). Culturally tailoring health care ensures practice is culturally safe and competent for the health professional, and leads to improved engagement and health related outcomes for consumers [39, 40]. Western health systems often focus on deficit-based approaches, for example: *you* have diabetes and therefore need nutrition and physical activity advice [30], in contrast to other cultural views such as Maaori, which encompasses pride in heritage, understanding kai / food and its connection to wider values and meanings [30]. Fear of judgement and shame may deter *whaanau* from reaching out for the care, support and information they need during pregnancy [24, 41] and postpartum [42, 43]. Therefore, culturally relevant communication strategies are important in engaging and supporting sustained behavioural change across generations [44].

The importance of *whaanau* / family to our participants (as described in theme three) reiterates that health care must be inclusive of a mother’s social supports. *Whaanau* / family-centred care is proficient in achieving favourable health outcomes, across both primary and secondary services [45]. Family-centred interventions improve wellbeing outcomes for indigenous children with some literature reviewed finding improved birth weights [46]reduced weight gain of children with BMI’s in the obese range [47] and improved immunisation and screening rates [48, 49]. Holistic care, which includes social supports (*whaanau* / family), are essential to the effective management of DiP and can have a long-lasting positive influence on infants and *whaanau* health and wellbeing [50].

### Study strengths and limitations

Equitable distribution of interviewee’s was one strength of this study. We interviewed equal numbers of Maaori and Pacific participants, who make up the majority of our population with diabetes, and experience a greater burden of adverse health outcomes due to diabetes in pregnancy. Furthermore, the team conducting the analysis were diverse in both ethnicity with representation across these ethnic groups, and multidisciplinary, including consumer and health care provider perspectives. Another strength lay in our approach, where we were able to re-present our initial codes and subthemes to participants, to receive feedback and ensure correct interpretation of their interviews before progressing the analysis. However, we acknowledge our results are reflective of the population we service in Counties Manukau, Aotearoa New Zealand; due to differences in communities and cultural needs may be less relevant to informing care in communities where there are lower number of Pacific, Maaori or Indian women, or where organised long-term post pregnancy support is in place. We also acknowledge the potential risk associated in our methodology; whereby potential participants were to be excluded if they were unable to conduct the interview in English. While these criteria did not impact any participant involvement, some participants may have been limited in their responses by their English vocabulary. Future studies into lived experience should look into the use of interpreting services and coding strategies to support interviews in the participants preferred language.

### Implications for practice

As Maaori, Pacific and Indian ethnicities make up the majority of women with diabetes in pregnancy in Counties Manukau, understanding what culturally safe, competent, and relevant care is, along with its delivery to these groups, is a priority to any new service.

The findings from this study were presented to the Children Affected by Diabetes in Pregnancy Model of Care Project Steering Group (see acknowledgements). This multi-disciplinary and multi-ethnic governance committee endorsed a continuity model of care design pitched by the Study Working Group. This model of care provides connection and navigation for both the mother, and family / *whaanau* to establish enablers, support overcoming barriers and support health promoting behaviours. The model of care would begin in pregnancy for women with a diagnosis of GDM or T2DM, and continue through the first 1–2 years of the infant’s life [51]. Connection would be offered on scheduled and as needed basis, aiming to meet women and whaanau where they are, with regards to their priorities and health journey. Support will be individualised to the whanau / family’s needs, medicated through either social media, phone / text, online or visits to the family home or in community clinics to promote family involvement. We suggest community-based health care worker/s, who are knowledgeable / experienced in, or who has a culturally similar background to users of our diabetes in pregnancy service, to optimise cultural safety and relevance. The healthcare worker/s are provided with education and training to promote what is “best practice” but are also provided with education and links to existing community, primary and secondary care services relevant areas of child and maternal health. The community-based health care worker therefore provides some individualised support and education but also supports the family to navigate existing health care supports within our community. We propose to pilot this service with a continuous evaluation programme in place, to allow adaptation and development of the service in response to feedback and needs. Assessing the impact on maternal wellbeing will be a central part of this evaluation.

## Conclusion

People who are affected by diabetes in pregnancy have ongoing physical, psychological and social needs. To our knowledge, this is the first study of the experiences of establishing infant feeding, complementary feeding (solids), sleep, physical activity, and screen time, amongst those who have had pregnancies complicated by diabetes, for the purpose of developing a new model of care. The findings of our analysis emphasis a need for a holistic, *whaanau*-centred approach to ensure optimal health and well-being outcomes for mothers, infants and *whaanau*. The findings of this study will inform a new targeted model of care to be piloted in 2024, for mothers, infants and *whaanau* affected by diabetes in pregnancy.

## Data Availability

The datasets used and analysed during this study are available from the corresponding author on reasonable request.

## Abbreviations

GDM: Gestational Diabetes Mellitus
T2DM: Type Two Diabetes Mellitus
DiP: Diabetes in Pregnancy

## References

[1] Schwarzenberg SJ, Georgieff MK, NUTRITION CON, Daniels S, Corkins M, Golden NH, Advocacy for Improving Nutrition in the First 1000 Days to Support Childhood Development and Adult Health. Pediatrics [Internet]., et al. 1;141(2):e20173716. Available from: 2018Feb. 10.1542/peds.2017-3716. 10.1542/peds.2017-3716 29358479

[2] Woo Baidal JA, Locks LM, Cheng ER, Blake-Lamb TL, Perkins ME, Taveras EM. Risk Factors for Childhood Obesity in the First 1,000 Days: A Systematic Review. Am J Prev Med [Internet]. 2016 Jun 1;50(6):761–79. Available from: 10.1016/j.amepre.2015.11.012 10.1016/j.amepre.2015.11.012 26916261

[3] Hawdon J. Babies born after diabetes in pregnancy: what are the short- and long-term risks and how can we minimise them? Best Pract Res Clin Obstet Gynaecol. 2011;25(1):91–104. 21237719 10.1016/j.bpobgyn.2010.10.005

[4] Yan J, Liu L, Zhu Y, Huang G, Wang PP. The association between breastfeeding and childhood obesity: a meta-analysis. BMC Public Health [Internet]. 2014;14(1):1267. Available from: 10.1186/1471-2458-14-1267 10.1186/1471-2458-14-1267 PMC4301835 25495402

[5] Hennessy M, Heary C, Laws R, Van Rhoon L, Toomey E, Wolstenholme H, et al. Policy Brief | The effectiveness of health professional‐delivered interventions during the first 1,000 days to prevent overweight/obesity in children: A systematic review. Obesity Reviews [Internet]. 2019;20(12):1691–707. Available from: 10.1111/obr.12924. 10.1111/obr.12924 31478333

[6] Woo Baidal JA, Locks LM, Cheng ER, Blake-Lamb TL, Perkins ME, Taveras EM. Risk Factors for Childhood Obesity in the First 1,000 Days: A Systematic Review. Am J Prev Med [Internet]. 2016 Jun 1;50(6):761–79. Available from: 10.1016/j.amepre.2015.11.012 10.1016/j.amepre.2015.11.012 26916261

[7] Moore T, Arefadib N, Deery A, West S. The First Thousand Days: an Evidence Paper. Centre for Community Child health. Parkville: Murdoch Children's Research Institute; 2017.

[8] Choudhury AA, Devi Rajeswari V. Gestational diabetes mellitus - A metabolic and reproductive disorder. Biomedicine & Pharmacotherapy [Internet]. 2021;143:112183. Available from: https://www.sciencedirect.com/science/article/pii/S0753332221009677 10.1016/j.biopha.2021.112183 34560536

[9] Schaefer-Graf U, Napoli A, Nolan CJ. Diabetes in pregnancy: a new decade of challenges ahead. Diabetologia [Internet]. 2018 May;61(5):1012–21. Available from: https://www.proquest.com/scholarly-journals/diabetes-pregnancy-new-decade-challenges-ahead/docview/1993310123/se-2?accountid=47386 10.1007/s00125-018-4545-y PMC6448995 29356835

[10] Te Whatu Ora Counties Manukau. Tuuranga Hauora o te Mana Waahine report/Division of Women’s Health report by Te Whatu Ora Counties Manukau - Issuu [Internet]. 2023 [cited 2023 Aug 26]. Available from: https://issuu.com/communicationsmiddlemore/docs/cmh_maternity_annual_report_2022_book_final_v5_web

[11] McKree Jansen R, Sundborn G, Cutfield R, Yu D, Simmons D. Ethnic inequity in diabetes outcomes-inaction in the face of need. Vol. 133, New Zealand Medical Journal. 2020. 33223543

[12] Shepard-Wipiiti T BL. The Economic and Social Cost of Type 2 Diabetes [resreport] [Internet]. 2021 [cited 2023 Jun 27]. Available from: https://healthierlives.co.nz/report-on-the-economic-and-social-cost-of-type-2-diabetes/

[13] Sise A, Donald S, Coppell KJ, Barson D, Crengle S, Parkin L, Are women with gestational diabetes being screened for type 2 diabetes following pregnancy? A nationwide retrospective cohort study in Aotearoa New Zealand. Diabetes Res Clin Pract [Internet]. 1;194. Available from: 2022Dec. 10.1016/j.diabres.2022.110139. 10.1016/j.diabres.2022.110139 36328213

[14] Ministry of Health. Diabetes in Pregnancy. Quick reference guide for health professionals on the screening, diagnosis and treatment of gestational diabetes in New Zealand. Wellington: Ministry of Health. 2014. Available from: https://www.tewhatuora.govt.nz/publications/diabetes-in-pregnancy.

[15] Manerkar K, Harding J, Conlon C, McKinlay C. Maternal gestational diabetes and infant feeding, nutrition and growth: a systematic review and meta-analysis. British Journal of Nutrition [Internet]. 2020/01/22. 2020;123(11):1201–15. Available from: https://www.cambridge.org/core/article/maternal-gestational-diabetes-and-infant-feeding-nutrition-and-growth-a-systematic-review-and-metaanalysis/20C2DCE802E96D330294D28ABF25E1A7 10.1017/S0007114520000264 31964432

[16] Chen KR, Yu T, Lien YJ, Chou YY, Kuo PL. Childhood neurodevelopmental disorders and maternal diabetes: A population-based cohort study. Dev Med Child Neurol [Internet]. 2023 Jul 1;65(7):933–41. Available from: 10.1111/dmcn.15488 10.1111/dmcn.15488 36541040

[17] Dennedy MC, Dunne F. The maternal and fetal impacts of obesity and gestational diabetes on pregnancy outcome. Best Pract Res Clin Endocrinol Metab [Internet]. 2010;24(4):573–89. Available from: https://www.sciencedirect.com/science/article/pii/S1521690X10000588 10.1016/j.beem.2010.06.001 20832737

[18] Shuffrey LC, Lucchini M, Morales S, Sania A, Hockett C, Barrett E, et al. Gestational diabetes mellitus, prenatal maternal depression, and risk for postpartum depression: an Environmental influences on Child Health Outcomes (ECHO) Study. BMC Pregnancy Childbirth [Internet]. 2022;22(1):758. Available from: 10.1186/s12884-022-05049-4 10.1186/s12884-022-05049-4 PMC9548153 36209070

[19] Gkiouleka A, Wong G, Sowden S, Bambra C, Siersbaek R, Manji S, et al. Reducing health inequalities through general practice. Lancet Public Health [Internet]. 2023;8(6):e463–72. Available from: https://www.sciencedirect.com/science/article/pii/S2468266723000932 10.1016/S2468-2667(23)00093-2 37244675

[20] Te One A, Clifford C. Tino Rangatiratanga and Well-being: Māori Self Determination in the Face of Covid-19. Frontiers in Sociology [Internet]. 2021;6. Available from: https://www.frontiersin.org/articles/10.3389/fsoc.2021.613340 10.3389/fsoc.2021.613340 PMC8022796 33869564

[21] Wilson D, Moloney E, Parr JM, Aspinall C, Slark J. Creating an Indigenous Māori-centred model of relational health: A literature review of Māori models of health. J Clin Nurs [Internet]. 2021 Dec 1;30(23–24):3539–55. Available from: 10.1111/jocn.15859 10.1111/jocn.15859 PMC8597078 34046956

[22] Teariki MA, Leau E. Understanding Pacific worldviews: principles and connections for research. Kōtuitui: New Zealand Journal of Social Sciences Online [Internet]. :1–20. Available from: 10.1080/1177083X.2023.2292268

[23] Hunt D, Lamb K, Elliott J, Hemmingsen B, Slama S, Scibilia R, A WHO key informant language survey of people with lived experiences of diabetes: Media misconceptions, values-based messaging, stigma, framings and communications considerations. Diabetes Res Clin Pract [Internet]., et al. 1;193. Available from: 2022Nov. 10.1016/j.diabres.2022.110109. 10.1016/j.diabres.2022.110109 36183868

[24] Jarvie R. Lived experiences of women with co-existing BMI≥30 and Gestational Diabetes Mellitus. Midwifery [Internet]. 2017;49:79–86. Available from: https://www.sciencedirect.com/science/article/pii/S0266613816303291 10.1016/j.midw.2016.12.009 28011058

[25] Rendle KA, Abramson CM, Garrett SB, Halley MC, Dohan D. Beyond exploratory: a tailored framework for designing and assessing qualitative health research. BMJ Open [Internet]. 2019 Aug 1;9(8):e030123. Available from: http://bmjopen.bmj.com/content/9/8/e030123.abstract 10.1136/bmjopen-2019-030123 PMC6720470 31462482

[26] Neubauer BE, Witkop CT, Varpio L. How phenomenology can help us learn from the experiences of others. Perspect Med Educ [Internet]. 2019;8(2):90–7. Available from: 10.1007/s40037-019-0509-2 10.1007/s40037-019-0509-2 PMC6468135 30953335

[27] Ware F, Breheny M, Forster M. Kaupapa Kōrero: a Māori cultural approach to narrative inquiry. AlterNative: An International Journal of Indigenous Peoples. 2017;5(14):117718011774481.

[28] Vaioleti TM. Talanoa Research Methodology: A Developing Position on Pacific Research. Waikato Journal of Education [Internet]. 2016 Sep 22;12(1). Available from: https://wje.org.nz/index.php/WJE/article/view/296

[29] Tapera R, Harwood M, Anderson A. A qualitative Kaupapa Māori approach to understanding infant and young child feeding practices of Māori and Pacific grandparents in Auckland, New Zealand. Public Health Nutr [Internet]. 2016/11/10. 2017;20(6):1090–8. Available from: https://www.cambridge.org/core/article/qualitative-kaupapa-maori-approach-to-understanding-infant-and-young-child-feeding-practices-of-maori-and-pacific-grandparents-in-auckland-new-zealand/FE726D26DD44A3F25B67F0B2008CA499 10.1017/S1368980016002950 PMC10261593 27829473

[30] Heke I, Rees D, Swinburn B, Waititi RT, Stewart A. Systems Thinking and indigenous systems: native contributions to obesity prevention. AlterNative: An International Journal of Indigenous Peoples [Internet]. 2018 Oct 24;15(1):22–30. Available from: 10.1177/1177180118806383

[31] Whitty-Rogers J, Cameron B, Caine V. An Indigenous and Western paradigm to understand gestational diabetes mellitus: Reflections and insights. Action Research. 2020Oct;13(21):147675032096082.

[32] Pogorzelska K, Chlabicz S. Patient Satisfaction with Telemedicine during the COVID-19 Pandemic—A Systematic Review. Int J Environ Res Public Health [Internet]. 2022;19(10). Available from: https://www.mdpi.com/1660-4601/19/10/6113 10.3390/ijerph19106113 PMC9140408 35627650

[33] Bradford BF, Cronin RS, Okesene-Gafa KA, Apaapa-Timu THS, Shashikumar A, Oyston CJ. Diabetes in pregnancy: Women’s views of care in a multi-ethnic, low socioeconomic population with midwifery continuity-of-care. Women and Birth [Internet]. 2024;37(3):101579. Available from: https://www.sciencedirect.com/science/article/pii/S1871519224000179. 10.1016/j.wombi.2024.01.005 38296743

[34] Okesene-Gafa KAM, Li M, McKinlay CJD, Taylor RS, Rush EC, Wall CR, et al. Effect of antenatal dietary interventions in maternal obesity on pregnancy weight-gain and birthweight: Healthy Mums and Babies (HUMBA) randomized trial. Am J Obstet Gynecol [Internet]. 2019 Aug 1;221(2):152.e1–152.e13. Available from: 10.1016/j.ajog.2019.03.003 10.1016/j.ajog.2019.03.003 30878323

[35] Mihrshahi S, Vaughan L, Fa’avale N, De Silva Weliange S, Manu-Sione I, Schubert L. Evaluation of the Good Start Program: a healthy eating and physical activity intervention for Maori and Pacific Islander children living in Queensland, Australia. BMC Public Health [Internet]. 2017;17(1):77. Available from: 10.1186/s12889-016-3977-x 10.1186/s12889-016-3977-x PMC5237210 28086843

[36] Singhal M, Telehealth OC, Technology for Diabetes in Pregnancy Clinics: Staff Perspectives from South Auckland, New Zealand. Int J Telemed Appl [Internet]. 1;2024(1):6429519. Available from: 2024Jan. 10.1155/2024/6429519. 10.1155/2024/6429519 PMC10957257 38516417

[37] Snelgrove-Clarke E, Grant S, Brenton-Peters J. Weight Management Guidelines for Patients With Gestational or Type 2 Diabetes During Pregnancy: Are the Current Guidelines Reflective of Shared Decision Making? The Diabetes Communicator. Spring edition 2020;18–20.

[38] Dahlberg H, Berg M, The lived experiences of healthcare during pregnancy, birth, and three months after in women with type 1 diabetes mellitus. Int J Qual Stud Health Well-being [Internet]. 1;15(1):1698496. Available from: 2020Jan. 10.1080/17482631.2019.1698496. 10.1080/17482631.2019.1698496 PMC6922046 31825747

[39] Stotz S, Moore K, Charron-Prochownik D, Fischl A, Spencer K, Mau M. A Qualitative Study on Preventing Gestational Diabetes in Native Hawaiian and Pacific Islander Adolescent Females: Perspectives from an Expert Panel of Health Care Providers. Hawaii J Health Soc Welf. 2023Jan;1(82):10–5. PMC9850826 36685779

[40] Oxlad M, Whitburn S, Grieger J. The Complexities of Managing Gestational Diabetes in Women of Culturally and Linguistically Diverse Backgrounds: A Qualitative Study of Women’s Experiences. Nutrients. 2023;15(4):1053. 36839411 10.3390/nu15041053 PMC9967365

[41] Sun S, Pellowski J, Pisani C, Pandey D, Go M, Chu M, et al. Experiences of stigma, psychological distress, and facilitative coping among pregnant people with gestational diabetes mellitus. BMC Pregnancy Childbirth [Internet]. 2023;23(1):643. Available from: 10.1186/s12884-023-05949-z 10.1186/s12884-023-05949-z PMC10486063 37679726

[42] Davidsen E, Maindal HT, Rod MH, Olesen K, Byrne M, Damm P, et al. The stigma associated with gestational diabetes mellitus: A scoping review. EClinicalMedicine [Internet]. 2022;52:101614. Available from: https://www.sciencedirect.com/science/article/pii/S2589537022003443 10.1016/j.eclinm.2022.101614 PMC9386490 35990581

[43] Incollingo Rodriguez AC, Smieszek SM, Nippert KE, Tomiyama AJ. Pregnant and postpartum women’s experiences of weight stigma in healthcare. BMC Pregnancy Childbirth [Internet]. 2020;20(1):499. Available from: 10.1186/s12884-020-03202-5 10.1186/s12884-020-03202-5 PMC7457255 32854654

[44] Faletau J, Nosa V, Dobson R, Heather M, McCool J. Falling into a deep dark hole: Tongan people’s perceptions of being at risk of developing type 2 diabetes. Health Expectations [Internet]. 2020 Aug 1;23(4):837–45. Available from: 10.1111/hex.13056 10.1111/hex.13056 PMC7495076 32441864

[45] McCalman J, Heyeres M, Campbell S, Bainbridge R, Chamberlain C, Strobel N, et al. Family-centred interventions by primary healthcare services for Indigenous early childhood wellbeing in Australia, Canada, New Zealand and the United States: a systematic scoping review. BMC Pregnancy Childbirth [Internet]. 2017;17(1):71. Available from: 10.1186/s12884-017-1247-2 10.1186/s12884-017-1247-2 PMC5320754 28222689

[46] Tursan d’Espaignet E, Measey ML, Carnegie MA, Mackerras D. Monitoring the ‘Strong Women, Strong Babies, Strong Culture Program’: The first eight years. J Paediatr Child Health [Internet]. 2003 Dec 1;39(9):668–72. Available from: 10.1046/j.1440-1754.2003.00272.x 10.1046/j.1440-1754.2003.00272.x 14629497

[47] Harvey-Berino J, Rourke J. Obesity Prevention in Preschool Native-American Children: A Pilot Study Using Home Visiting. Obes Res [Internet]. 2003 May 1;11(5):606–11. Available from: 10.1038/oby.2003.87 10.1038/oby.2003.87 12740449

[48] Black AP, Daniel M, Esterman A, Karschimkus CS, Morris P, O’Dea K, et al. Nutritional impacts of a fruit and vegetable subsidy programme for disadvantaged Australian Aboriginal children. British Journal of Nutrition [Internet]. 2013/06/07. 2013;110(12):2309–17. Available from: https://www.cambridge.org/core/product/75D02DA4BD2318B405C82283E19587E3 10.1017/S0007114513001700 23742751

[49] Poole N. Evaluation Report of the Sheway Project for High-Risk Pregnant and Parenting Women [Internet]. British Columbia Centre of Excellence for Women’s Health. 2000. Available from: http://bccewh.bc.ca/wp-content/uploads/2012/05/2000_Evaluation-Report-of-the-Sheway-Project.pdf.

[50] Nielsen KK, Kapur A, Damm P, de Courten M, Bygbjerg IC. From screening to postpartum follow-up – the determinants and barriers for gestational diabetes mellitus (GDM) services, a systematic review. BMC Pregnancy Childbirth [Internet]. 2014;14(1):41. Available from: 10.1186/1471-2393-14-41 10.1186/1471-2393-14-41 PMC3901889 24450389

[51] Eades CE, France EF, Evans JMM. Postnatal experiences, knowledge and perceptions of women with gestational diabetes. Diabetic Medicine [Internet]. 2018 Apr 1;35(4):519–29. Available from: 10.1111/dme.13580 10.1111/dme.13580 29338094

